# The influence of curcumin additives on the viability of probiotic bacteria, antibacterial activity against pathogenic microorganisms, and quality indicators of low-fat yogurt

**DOI:** 10.3389/fnut.2023.1118752

**Published:** 2023-04-03

**Authors:** Magdalena Buniowska-Olejnik, Jakub Urbański, Artur Mykhalevych, Pawel Bieganowski, Agata Znamirowska-Piotrowska, Miroslava Kačániová, Maciej Banach

**Affiliations:** ^1^Department of Dairy Technology, Institute of Food Technology and Nutrition, University of Rzeszów, Rzeszów, Poland; ^2^Food Studies, SWPS University, Warsaw, Poland; ^3^Dairy Biotechnologies Ltd., Puławy, Poland; ^4^Department of Milk and Dairy Products Technology, Educational and Scientific Institute of Food Technologies, National University of Food Technologies, Kyiv, Ukraine; ^5^Department of Experimental Pharmacology, Mossakowski Medical Research Institute, Polish Academy of Sciences, Warsaw, Poland; ^6^Faculty of Horticulture and Landscape Engineering, Institute of Horticulture, Slovak University of Agriculture, Nitra, Slovakia; ^7^Department of Bioenergy, Food Technology and Microbiology, Institute of Food Technology and Nutrition, University of Rzeszow, Rzeszów, Poland; ^8^Department of Preventive Cardiology and Lipidology, Medical University of Łódź, Łódź, Poland; ^9^Cardiovascular Research Centre, University of Zielona Góra, Zielona Góra, Poland; ^10^Department of Cardiology and Adult Congenital Heart Diseases, Polish Mother’s Memorial Hospital Research Institute (PMMHRI), Łódź, Poland

**Keywords:** antimicrobial properties, dairy products, nutraceuticals, curcumin, innovative technology

## Abstract

Curcumin is a nutraceutical with unique anti-inflammatory, anti-oxidative, and antimicrobial properties. In this study, we aimed to examine the advantages of the use of water dispersible and highly bioavailable form of standardized turmeric extract (*Curcuma longa* L*.*)—NOMICU® L-100 (N) in the formulation of probiotic yogurt in comparison with the standard turmeric extract (TE). The antimicrobial activity of both supplements was studied and compared in the context of gram-positive and gram-negative bacteria, yeasts, and fungi. The N maintains the level of *Bifidobacterium animalis* subsp. *lactis* BB-2 in yogurt at the recommended level (7–9 log CFU/g) throughout the storage period. NOMICU® L-100 also has a higher inhibitory capacity for the growth of yeast and fungi. The evaluation of quality indicators of yogurt with N and TE at the level of 0.2% proves that yogurt with N has original taste properties. A lower degree of syneresis was noted for yogurt with TE (0.2%), but its sensory properties are unacceptable to the consumer due to the appearance of a bitter taste. In conclusion, based on the obtained results, it has been proven that the use of NOMICU® L-100 (0.2%) in the composition of yogurt provides a product of functional direction with stable quality and safety indicators, which can be stored for at least 28 days.

## 1. Introduction

Fermented milk drinks are dairy products of a liquid or semi-liquid consistency obtained by fermentation of a milk mix with special microorganisms that are part of starter preparations. Fermented milk drinks are classified into three types based on their shelf life: fresh fermented milk drinks with a short shelf life, those with an extended shelf life, and thermalized fermented milk drinks (1, 2). At the same time, since fermented milk products are perishable due to danger from fungi and yeast, their long-term suitability might be a problem. The process of thermalizing the mixture, which involves processing at a temperature of 60°C–70°C with a holding period of at least 30 min, is the most popular way to increase the shelf life of fermented milk products (3). This method, however, diminishes the sensory characteristics of the product during storage and may contribute to the partial denaturation of the milk proteins. Other methods may include the selection of special types of packaging (4, 5), the use of high-pressure processing (6), excess irradiation (7), high-temperature processing (2, 8), thermosonication treatment (9), and ozone treatment (10). The main problem with such methods is their negative impact on the microorganisms included in the fermentation preparations, in particular on their viability (11, 12).

For the mass production of fermented milk products, it is also common to use preservatives, which stop the decomposition processes that occur in non-living cells and reduce the activity of metabolic processes in living cells, thereby ensuring the product’s long-term storage (13, 14). In the case of choosing preservatives, manufacturers should always consider the mechanism of action because each preservative acts selectively on a certain type of microorganism. In the absence of a scientific explanation for the selection of preservatives, they may significantly inhibit the activity of the lactic acid microflora of fermented milk drinks, even at minimal doses (14). In the dairy industry, the most well-known preservatives are potassium sorbate, benzoic acid, and sorbic acid (15).

There are many studies available on the treatment of dairy products with sorbate (16–18), which indicates that *Aspergillus* spp., *A. parasiticus*, and *Penicillium camemberti* are inhibited by a concentration of 500 mg/kg of sorbate. Furthermore, adding potassium sorbate to yogurt at concentrations of 0.5%, 0.1%, and 0.2% inhibited yeast and fungi levels, with normal characteristic properties extending more than 14 days (19).

Natural preservatives include lactic acid microorganisms, which, during the fermentation of milk, produce antimicrobial metabolites, in particular, organic acids, such as propionic, acetic, and lactic acids (20). They create an unfavorable environment for the development of pathogenic microorganisms (21). Lactic acid bacteria are a diverse group of beneficial bacteria that have been unwittingly used by humans for thousands of years as starter cultures for the preparation of a variety of fermented dairy products (22), including yogurt, kefir, sour cream, baked cultured milk, acidophilic milk, cultured milk, ayran, koumiss, and others.

Increasing consumer interest in natural products that do not contain artificially synthesized additives has led to a renewed interest in the use of natural antimicrobials for food products, in particular, fermented milk drinks (23, 24). Thus, it is advisable to search for natural ingredients that, due to their properties and when combined with lactic acid microorganisms, may effectively stop the growth of pathogenic microorganisms and extend the fermented milk drink’s shelf life. Curcumin is a well-known naturally occurring nutraceutical with potent antimicrobial activity (25, 26). However, research on food products containing curcumin is limited due to its low native solubility in water and, thus, low bioavailability. Turmeric extract standardized for the 95% of curcuminoids content has very low water solubility estimated at 3.12 mg/L at 25°C (27) and is insoluble in unheated oil (28).

Moreover, the turmeric extract in its commercially available form has a hot-burning taste and a pungent odor, which may adversely affect the sensory qualities of the products. Due to its low solubility, the curcuminoid extract, when added to dairy products, both at the stage before and after fermentation, might form precipitates. Encapsulation is one of the proposed methods for increasing the bioavailability of curcumin (25), for which a scientific approach to selecting an encapsulating agent that will contribute to the formulation of a bioactive complex with curcumin in essential. If introduced into industrial practice, due to legal conditions, the food additive may not contain substances subject to restrictions on the scope of use and permissible concentrations, which excludes or limits the use of some formulations available on the market due to the content of non-GRAS (Generally Regarded As Safe) substances. Due to the sensory parameters, it is also advisable to use formulations based on highly purified curcuminoid extracts with a neutral taste and smell—considering the recommended daily doses in a safe range below 3 mg/kg b. w. (29).

Milk, and even more products fermented by lactic acid microorganisms, is one of the most promising food systems capable of transmitting the antioxidant properties of curcumin (30). Scientists have reported that milk proteins also show a synergistic effect with the encapsulating agent, as they possess pronounced structural and surface-active properties that stimulate the process of transport of biologically active substances (31). In addition, milk proteins can form covalent or electrostatic complexes with molecules of interest and capture biologically active substances by forming gels (32).

The complex of the bioactive form of curcumin and milk proteins is able not only to provide preventive direction to the fermented milk drink but also to show a synergistic effect together with lactic acid bacteria, thereby increasing their vital activity (33). Thus, Abdelrazik and Fouad M. F. Elshaghabee (29) reported that the addition of curcumin at different concentrations led to a significant increase in antibacterial activity against gram-positive bacteria *B. subtilis* and *S. aureus*, as well as gram-negative *E. coli* and *P. fluorescens* (30). Importantly, the antibacterial activity in fermented soy milk was significantly higher than that of non-fermented soy milk, which is attributed to the additional effect of acetic acid produced by lactic acid microorganisms (34). It was found that curcumin can promote the increase of viable cells of various lactic acid bacteria, such as *Lacticaseibacillus casei, Lactiplantibacillus plantarum* subsp*. plantarum, Bifidobacterium animalis* subsp. *lactis* BB-12, which is confirmed by the amount of probiotics at the end of the storage period that is higher than the recommended one (10^6^ CFU/g). The antibacterial activity of various probiotic strains plays an important role in increasing the shelf life of various types of food products. Their combination with a bioavailable form of curcumin, innovative packaging methods, and the selection of raw materials with a high degree of microbiological purity can ensure the production of a prophylactic fermented milk drink with an extended shelf life without the use of traditional artificially synthesized additives that have a negative effect on the human body.

Thus, we aimed to investigate the effect of the bioavailable form of curcumin on the viability of probiotic bacteria, antibacterial activity against pathogenic microorganisms, and quality indicators of yogurt. Specifically, our purpose was to determine and compare the antimicrobial activity of selected curcumin preparations (turmeric extract, NOMICU® L-100), to test the hypothesis regarding the ability of curcumin to influence the viability of probiotic cultures using the example of *Bifidobacterium animalis* subsp. *lactis* BB-12, and to suppress the development of coliforms, yeasts, and fungi in order to select the best doses of curcumin preparations (turmeric extract, NOMICU® L-100) in yogurt formulations for further research, and finally to investigate the effect of selected doses of turmeric extract and NOMICU® L-100 on the physicochemical indicators of yogurt (active and titrated acidity, the amount of lactic acid, the degree of syneresis and color indicators), as well as to evaluate sensory indicators of the experimental samples during the storage period of 28 days.

## 2. Materials and methods

### 2.1. Materials

For the experiment, two forms of curcumin were used. The first, NOMICU® L-100 (N), is a water-dispersible, highly purified, and highly bioavailable form of standardized turmeric extract *Curcuma longa* L. (TE) with additives, permitted in food supplements (compliant with regulation (EС) № 1333/2008 of the European Parliament and of the Council of 16 December 2008 on food additives, GRAS—Generally Regarded as Safe). The N is a proprietary formulation containing a full spectrum of curcuminoids for use as an active ingredient in dietary and food supplements, functional beverages and dairy products, cosmetics, and MCT oils. The second one is TE, which contains 95% of curcumin. For yogurt production, the culture starters YC-X16 (Chr. Hansen, Hvidovre, Denmark; consists of *Streptococcus thermophilus* and *Lactobacillus delbrueckii* subsp. *bulgaricus*) and BB-12 (Chr. Hansen, Hvidovre, Denmark; consists of *Bifidobacterium animalis* subsp. *lactis* BB-12) were used.

### 2.2. Preparation of yogurt

The milk with 3.2% of fat with skimmed milk powder of 1.5% was pasteurized at 85°C for 30 min, then cooled to 40°C, and divided into groups including C—control milk, TE—sample with turmeric extract addition of curcumin form; N—turmeric extract (*Curcuma longa* L.)—NOMICU® L-100. Next, the milk was inoculated with a single starter culture YC-X16 (Chr. Hansen, Hvidovre, Denmark; consists of *Streptococcus thermophilus* and *Lactobacillus delbrueckii* subsp. *bulgaricus*) and *Bifidobacterium animalis* subsp. *lactis* BB-12 (Chr. Hansen, Hvidovre, Denmark). An activated starter was made by inoculation of milk with DVS cultures. Each batch of milk was inoculated with a previously activated starter culture (in the form of bulk activated at 40°C for 10 h). Inoculated milk was stirred and fermented at 40°C for 10 h. After fermentation, curcumin in different forms (TE and N) was added in amounts of 0%, 0.10%, 0.15%, 0.20%, and 0.25%, respectively. The final products were stirred and poured into 100-ml plastic cups and cooled to 5°C.

### 2.3. Methods

#### 2.3.1. Antimicrobial activity

For antimicrobial analyses of clear powder, the well agar diffusion method was used. Antibacterial activities of N and TE were evaluated using the well diffusion method on Mueller-Hinton agar (MHA, Oxoid, Basingstoke, United Kingdom) for bacteria and Sabouraud Dextrose Agar (SDA, Oxoid, Basingstoke, United Kingdom) for yeast and microscopic fungi. The inhibition zones were reported in millimeters (mm). Gram-positive (G^+^, *Staphylococcus aureus* subsp*. aureus* CCM 2461, *Listeria monocytogenes* CCM 4699, and *Streptococcus pneumoniae* CCM 4501) and gram-negative (G^−^, *Escherichia coli* CCM 3988, *Pseudomonas aeruginosa* CCM 1959, *and Yersinia enterocolitica* CCM 5671) bacteria, yeasts (*Candida albicans* CCM 8186, *C. glabrata* CCM 8270, *C. krusei* CCM 8271, and *C. tropicalis* CCM 8223), fungi (*Penicillium chrysogenum, P. brevicompactum*, *P. italicum*, and *P. aurantiogriseum*) were used as references for the antimicrobial assay of N and TE. Bacteria and yeasts were purchased from the Czech Collection of Microorganisms (Brno, Czech Republic). After 24 h, single colonies on agar plates were used to prepare the microbial suspension with a turbidity of 0.5 McFarland [equal to 1.5 × 10^8^ colony-forming units (CFU/mL)]. The turbidity of the bacterial suspension was measured at 600 nm. MHA agar plates were inoculated with bacterial strain under aseptic conditions, and wells (*d* = 6 mm) were filled with 50 μL of the test samples and incubated at 37°C for 24 h. Microscopic fungi and yeasts were evaluated on SDA at 25°C for 24 h. After the incubation period, the diameter of the growth inhibition zones was measured in triplicate.

The Minimum Inhibitory Concentrations (MIC) of bacteria and yeasts were determined using the agar microdilution method. The inoculum was cultured for 24 h in Mueller-Hinton Broth (MHB, Oxoid, Basingstoke, United Kingdom) at 37°C for bacteria and Sabouraud Dextrose Broth (SDB, Oxoid, Basingstoke, United Kingdom) at 25°C for yeast. A sample of 100 μL of nutrient medium and 50 μL of inoculum with an optical density of 0.5 McFarland standard were applied to a 96-well microtiter plate. Subsequently, N and TE were prepared by serial dilution to a concentration range of 11 mg/ml to 0.010 mg/ml in MHB / SDB and mixed thoroughly with bacterial and yeast inoculum in the wells. The prepared 96-well microtiter plates were measured at 570 nm with a Glomax spectrophotometer (Promega Inc., Madison, WI, USA) at 0 h. Subsequently, the bacterial samples were incubated at 37°C for 24 h. Yeast samples were incubated at 25°C for 24 h and measured again. MHB/SDB with essential oil was used as a negative control, and MHB/SDB with inoculum was used as a positive control for maximal growth.

#### 2.3.2. Microbiological analyses

To conduct the study, 5 g of each sample of yogurt was diluted in 45 ml of physiological solution. The microbiological analysis was carried out at the following conditions: total number of microorganisms on PCA (Plate Count Agar, Oxoid, Basingstoke, United Kingdom) at 30°C for 48–72 h, number of coliforms bacteria on VRBL agar (Violet Red Bile agar with Lactose, Oxoid, Basingstoke, United Kingdom) at 37°C for 24–48 h, number of lactic acid bacteria on MRS (De Man Rogosa Sharpe, Oxoid, Basingstoke, United Kingdom), MSE (Mayeux, Sandine & Elliker, Oxoid, Basingstoke, United Kingdom) agars at 37°C for 48–72 h, and microscopic fungi and yeasts on MEA (Malt Extract agar, Oxoid, Basingstoke, United Kingdom) at 25°C for 5 days. The studies were carried out using the plate method with M17 agar (Biocorp, Warsaw, Poland) for *Streptococcus* and MRS for *Lacticaseibacillus* and *Bifidobacterium* (Biocorp, Warsaw, Poland). *Lacticaseibacillus* and *Bifidobacterium* were cultivated in microaerophilic conditions with 5% CO_2_ at 37°C. The identification of *Streptococcus* and *Bifidobacterium* was performed using MALDI-TOF MS.

### 2.4. Indicators of quality

#### 2.4.1. Acidity measurement

The pH value in milk after fermentation was determined by pH-meter FiveEasy (Mettler Toledo, Greifensee, Switzerland) using InLab®Solids Pro-ISM electrode (Mettler Toledo, Greifensee, Switzerland). Lactic acid content was determined according to Jemaa et al. (35). Fermented milk samples were titrated with 0.1 M NaOH in the presence of phenolphthalein as an indicator. Lactic acid content was expressed as g lactic acid L^−1^.

#### 2.4.2. Evaluation of color

The color was determined by a colorimeter (Precision Colorimeter, Model NR 145, Shenzhen, China) using the CIE LAB system. The following parameters were determined: L*—as lightness (from 0—black to 100—white), a*—as color from red (+) to green (−), b*—as color from yellow (+) to blue (−), C—as color purity and intensity, and h—as color hue. Before measurement, the device was calibrated on a white reference standard.

#### 2.4.3. Syneresis

Syneresis was determined by the centrifuge method using the Laboratory Refrigeration Centrifuge LMC-4200R (Biosan SIA, Riga, Latvia) according to Lopez-Santamarina et al. (36) method with modifications: 10 g of product was transferred into a 50 ml plastic tube and centrifuged at 1,789 × g for 10 min, temperature 19 ± 0.1°C. The separated whey was weighed and converted to percentages.

#### 2.4.4. Sedimentation

The stability of the aqueous dispersion of curcumin was measured in the sedimentation assay using a centrifuge MPW 351R with rotor no. 12177. TE and N were dispersed in 50 ml of distilled water and centrifuged in falcon tubes at 226 g for 10 min, at 19 ± 1 °C. Absorbance of the supernatant was measured at 450 nm on a spectrophotometer, (Helios-Beta, Unicam).

Sedimentation (%) was calculated using the formula:


$$\mathrm{Sedementation}=\left(1-\frac{\mathrm{after centrifugation}}{\mathrm{before centrifugation}}\;\right)\times 100$$

#### 2.4.5. Sensory evaluation

The sensory evaluation was carried out by a trained panel of experts for probiotic yogurt with curcumin (TE, N) and without curcumin at the 7th, 14th, 21st, and 28th days of refrigerated storage. Parameters were evaluated on a 9-point scale (from 1 = undetectable to 9 = very intense). The following parameters were evaluated: milky–creamy taste, sour taste, sweet taste, the taste of the additive, off-taste, fermentation odor, odor of the additive, and off-odor.

#### 2.4.6. Statistical analysis

The analysis of variance (ANOVA) was performed using STATISTICA 13 software. Test significance was set at *p* ≤ 0.05. Data are expressed as mean with standard deviations (±SD), and differences between groups were assessed using Tukey’s test. All the analysis was done in triplicate.

## 3. Results

### 3.1. Antimicrobial activity

The analysis of the antimicrobial activity of TE and N was carried out using G^+^ and G^−^ bacteria, yeasts, and mycelial fungi. TE and N show different antimicrobial activity against the studied microorganisms (Table 1). Thus, N has the highest antimicrobial activity against G^+^ bacteria with an inhibition zone of 8.00 ± 0.02 mm (*S. pneumoniae*), G^−^ bacteria with an inhibition zone of 3.00 ± 0.02 and 5.00 ± 0.02 mm (*P. aeruginosa* and *Y. enterocolitica*, respectively), and fungi with an inhibition zone of 7.00 ± 0.02 and 6.00 ± 0.04 mm (*P. italicum* and *P. aurantiogriseum*, respectively). At the same time, for TE, the highest antimicrobial activity was observed against G^+^ bacteria with an inhibition zone of 8.00 ± 0.02 and 9.00 ± 0.01 mm (*S. aureus* and *L. monnocytogenes*, respectively), G bacteria with an inhibition zone of 2.00 ± 0.01 mm (*E. coli*), yeast with an inhibition zone from 5.00 ± 0.02 mm (*C. albicans*) to 7.00 ± 0.02 mm (*C. tropicalis*), and microscopic mycelial fungi from 6.00 ± 0.03 mm (*P. italicum*) to 9.00 ± 0.01 mm (*P. chrysogenum*). The conducted MIC test for N revealed the lowest value for MIC 50 (0.09 mg/ml) for *C. tropicalis* and G^+^ bacterium (*S. pneumoniae*) and the highest for MIC 50 (3.60 mg/ml) and MIC 90 (4.20 mg/ml) for G^+^ bacterium (*S. aureus*). For TE, the test showed the lowest value of MIC 50 (0.06 mg/ml) and MIC 90 (0.09 mg/ml) for the G^+^ bacterium (*Streptococcus pneumoniae*) and the highest for MIC 50 (3.60 mg/ml) and MIC 90 (4.20 mg/ml) for G^+^ bacteria (*S. aureus*).

**Table 1:** Antimicrobial activity of TE and N.

Microorganism	Zone of inhibition, mm		MIC 50, mg/mL		MIC 90, mg/mL	
	TE	N	TE	N	TE	N
Gram-positive bacteria						
*Staphylococcus aureus*	8.00^b^ ± 0.02	6.00^a^ ± 0.03	3.60	3.60	4.20	4.20
*Listeria monnocytogenes*	9.00^b^ ± 0.01	7.00^a^ ± 0.02	1.80	1.80	2.50	2.50
*Streptococcus pneumoniae*	7.00^a^ ± 0.03	8.00^b^ ± 0.02	0.06	0.60	0.09	0.09
Gram-negative bacteria						
*Escherichia coli*	2.00^b^ ± 0.01	0.00^a^ ± 0.00	1.80	1.80	2.50	2.50
*Pseudomonas aeruginosa*	1.00^a^ ± 0.02	3.00^b^ ± 0.02	0.90	0.90	1.10	1.10
*Yersinia enterocolitica*	3.00^a^ ± 0.03	5.00^b^ ± 0.02	1.80	0.90	2.50	1.10
Yeasts						
*Candida albicans*	5.00^b^ ± 0.02	4.00^a^ ± 0.02	0.90	0.46	1.10	0.50
*Candida glabrata*	6.00^b^ ± 0.04	5.00^a^ ± 0.02	0.46	1.80	0.53	2.50
*Candida krusei*	6.00^b^ ± 0.02	5.00^a^ ± 0.04	–	–	–	–
*Candida tropicalis*	7.00^b^ ± 0.02	6.00^a^ ± 0.01	0.46	0.06	0.53	0.09
Fungi						
*Penicillium chrysogenum*	9.00^b^ ± 0.01	8.00^a^ ± 0.02	–	–	–	–
*Penicillium brevicompactum*	8.00^b^ ± 0.02	6.00^a^ ± 0.02	–	–	–	–
*Penicillium italicum*	6.00^a^ ± 0.03	7.00^b^ ± 0.02	–	–	–	–
*Penicillium aurantiogriseum*	5.00^a^ ± 0.02	6.00^b^ ± 0.04	–	–	–	–

^a–c^Mean values denoted by different letters in the column differ significantly at *p* ≤ 0.05.

### 3.2. Microbiological analyses

**Figure 1:**
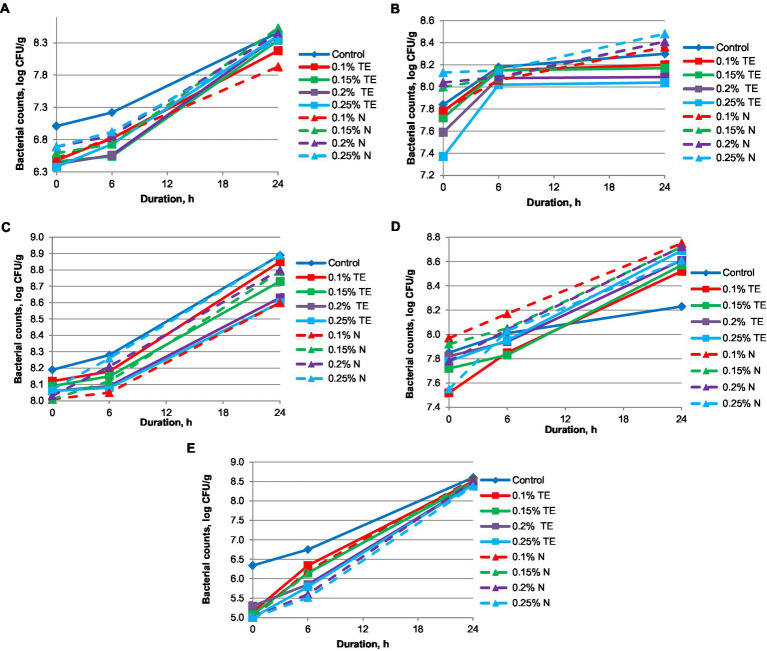
Growth dynamics of probiotic cultures. **(A)** yogurt cultures (*Streptococcus thermophilus* and *Lactobacillus delbrueckii* subsp. *bulgaricus)*, **(B)** *Lactobacillus acidophilus* LA-5®, **(C)** *Lacticaseibacillus casei* 431®, **(D)** *Lactobacillus johnsonii* La 1, **(E)** *Bifidobacterium animalis* subsp. *lactis* BB-12.

The results of growth dynamics of bacteria cultures: a—yogurt cultures (*Lactobacillus delbrueckii* subsp. *bulgaricus* and *Streptococcus thermophilus*), b—*Lactobacillus acidophilus* LA-5® (Chr. Hansen, Hvidovre, Denmark), c—*Lacticaseibacillus casei* 431® (Chr. Hansen, Hvidovre, Denmark), d—*Lactobacillus johnsonii* La 1 (Chr. Hansen, Hvidovre, Denmark), e—*Bifidobacterium animalis* subsp. *lactis* BB-12 (Chr. Hansen, Hvidovre, Denmark) are shown in Figure 1. The dynamics of the growth of microorganisms for 24 h indicates that N more actively stimulates the development of yogurt cultures*, Lactobacillus acidophilus, Lactobacillus johnsonii,* and *Lacticaseibacillus casei*. At the same time, for *Bifidobacterium animalis* subsp. *lactis* BB-12, the same effect is observed for both supplements (N and TE). For the yogurt cultures, the activity of N (0.15%–0.25%) is observed from the first hours, whereas for TE (0.15%–0.25%), the active growth of this strain begins after the 6th hour and reaches an insignificantly smaller number of microorganisms in comparison to N (log CFU/g 8.34–5.53). For *Lactobacillus acidophilus*, the growth of bacteria in the case of N (0.15%–0.25%) is dynamic, while for TE (0.1%–0.25%), there is a sharp increase in the number of viable cells of the probiotic within the first 6 h, leading to a plateau, where the number of probiotic bacteria does not change in a significant manner within the next 18 h. The dynamics of the growth of *Lacticaseibacillus casei* microorganisms are somewhat different. In Figure 1, it can be seen that the dose of TE in the concentration of 0.1% is quite sufficient to achieve a sufficiently high result (8.85 log CFU/g), which is lower only than the sample with N (8.89 log CFU/ g) for doses of 0.25%. An increase in the dose of N from 0.1% to 0.25% reduces the activity of the development of *Lactobacillus johnsonii* microorganisms, and for TE, on the contrary, it is slightly increased. Despite this, the total number of probiotic microorganisms is the highest for N (8.75 log CFU/g) at doses of 0.1%. The effect of TE and N on *Bifidobacterium animalis* subsp. *lactis* BB-12 (Chr. Hansen, Hvidovre, Denmark) is marked by stable dynamic growth. TE (0.1%) within 18–20 h shows the most active effect on probiotic microorganisms, which then decreases slightly and gives way to N (0.1%–0.15%).

The results of the microbiological analysis of yogurt samples during the 28 days of storage are shown in Тable 2. The number of viable bacteria *Bifidobacterium animalis* subsp. *lactis* BB-12 during the first 3 weeks of storage for all samples was within the limits recommended for functional fermented milk products (7–9 log CFU/g) (37). On the 28th day, for 0.25% N, their number decreased to 6.7 log CFU/g, which still exceeds the minimum permissible level for fermented milk products (6 log CFU/g) (38). At the same time, for TE (0.1%–0.25%) at the end of the storage period, the number of probiotic bacteria was lower than recommended (6 log CFU/g), which may be associated with a higher accumulation of lactic acid (39) than in the case of N. Representatives of coliforms in yogurt samples were not detected during the entire storage period, which is a consequence of an acidic environment, unfavorable for the appearance and development of this representative of the pathogenic microflora. The count of yeasts and microfungi indicates that N has a more pronounced effect on them than TE. Even a minimum dose of 0.1% N reduces their number during 3 weeks of storage compared to the control. An increased amount of this additive (0.2%–0.25%) completely suppresses their appearance during the 1st week of storage. TE also slightly suppresses the development of these microorganisms. However, this effect is not as pronounced as for N. Probably, N exhibits a synergistic effect together with the probiotic culture of *Bifidobacterium animalis* subsp. lactis BB-12, which is a consequence of not only the antimicrobial effect on pathogenic bacterial strains but also the survival of the yogurt starter strain (40). Although TE also exhibits antimicrobial activity against yeasts, microfungi, and coliforms, it adversely affects the development of the probiotic culture, which significantly reduces the possible shelf life of the product. Thus, yogurt samples with a dosage of N and TE at the level of 0.2% were selected for further investigation of quality indicators as the most appropriate due to the detection of the antibacterial effect and simultaneous support of the survival of the probiotic strain (Table 2).

**Table 2:** Microbial count (log CFU/g) of yogurt samples.

Sample	Storage time (days)	Values			
		Coliform count	BB-12 count	Yeasts count	Fungi count
Control	1	ND	>7.7	>7.7	6.0
	7	ND	>7.7	>7.7	6.2
	14	ND	>7.7	>7.7	6.4
	21	ND	>7.7	>7.7	6.8
	28	ND	>7.7	>7.7	>7.7
0.10% N	1	ND	>7.7	5.4	5.3
	7	ND	>7.7	5.5	5.5
	14	ND	>7.7	5.8	5.8
	21	ND	>7.7	7.0	5.8
	28	ND	>7.7	>7.7	>7.7
0.15% N	1	ND	>7.7	5.3	5
	7	ND	>7.7	5.4	5.3
	14	ND	>7.7	5.5	5.3
	21	ND	>7.7	7.0	5.7
	28	ND	>7.7	7.7	6.3
0.20% N	1	ND	>7.7	5.0	ND
	7	ND	>7.7	5.3	ND
	14	ND	>7.7	5.5	5.2
	21	ND	>7.7	6.9	5.7
	28	ND	>7.7	7.7	6.2
0.25% N	1	ND	>7.7	5.0	ND
	7	ND	>7.7	5.2	ND
	14	ND	>7.7	5.5	5.2
	21	ND	>7.7	6.2	5.4
	28	ND	6,69	6.7	6.2
0.10% TE	1	ND	>7.7	7.2	5.6
	7	ND	>7.7	>7.7	5.7
	14	ND	>7.7	>7.7	6.3
	21	ND	>7.7	>7.7	6.8
	28	ND	5,3	>7.7	>7.7
0.15% TE	1	ND	>7.7	6.3	5.5
	7	ND	>7.7	6.7	5.8
	14	ND	>7.7	6.9	6.3
	21	ND	>7.7	7.1	6.8
	28	ND	5	>7.7	7.0
0.20% TE	1	ND	>7.7	5.5	5.3
	7	ND	>7.7	5.5	5.8
	14	ND	>7.7	6.9	6.2
	21	ND	>7.7	7.0	6.6
	28	ND	4,7	7.7	6.8
0.25% TE	1	ND	>7.7	5.2	ND
	7	ND	>7.7	5.5	ND
	14	ND	>7.7	6.7	5.3
	21	ND	>7.7	6.9	5.9
	28	ND	4,5	7.1	6.7

ND—not detected.

### 3.3. Indicators of quality

Active acidity for both TE and N samples was slightly lower than in the control sample (Table 3). Thus, its significant decrease was observed during the first 2 weeks of storage, which corresponds to the active phase of the transformation of lactose into lactic acid. During the 3rd and 4th weeks of storage, the growth of acidity for N slowed down more than for TE, but in both cases, it was lower than in the control sample. At the same time, for all samples, the active acidity corresponded to the regulated limits of pH 4.0–4.6 (41) during the entire storage period of 28 days. The dynamics of changes in active acidity are consistent with the data obtained during the measurement of titrated acidity and lactic acid. It can be concluded that the addition of both TE and N does not significantly affect the change in product acidity.

**Table 3:** Indicators of quality of yogurt samples.

Properties	Storage time (days)	Values		
		C	0.20% TE	0.20% N
pH	1	4.59^bD^ ± 0.01	4.58^ab^D ± 0.01	4.57^aD^ ± 0.01
	7	4.38^abC^ ± 0.02	4.36^aB^ ± 0.01	4.40^bC^ ± 0.01
	14	4.18^aA^ ± 0.02	4.24^bA^ ± 0.01	4.26^bA^ ± 0.01
	21	4.26^aB^ ± 0.02	4.31^bC^ ± 0.02	4.35^cB^ ± 0.01
	28	4.28^aB^ ± 0.02	4.33^bB^ ± 0.02	4.37^cB^ ± 0.01
Total acidity (the titratable acidity), g L^−1^	1	36.53^abA^ ± 0.61	37.01^bA^ ± 0.21	35.20^aA^ ± 0.40
	7	41.20^abB^ ± 1.13	42.20^bC^ ± 0.28	40.40^aB^ ± 0.01
	14	43.60^bC^ ± 0.57	41.00^aB^ ± 0.28	40.80^aC^ ± 0.01
	21	44.00^bD^ ± 0.01	44.40^cD^ ± 0.01	42.20^aD^ ± 0.28
	28	45.00^bD^ ± 0.01	45.40^c^ ± 0.01	43.20^aE^ ± 0.28
Lactic acid, g L^−1^	1	0.77^bA^ ± 0.01	0.75^aA^ ± 0.01	0.75^aA^ ± 0.01
	7	0.85^aB^ ± 0.01	0.88^bB^ ± 0.02	0.87^bB^ ± 0.01
	14	0.91^aC^ ± 0.02	0.92^aC^ ± 0.01	0.91^aC^ ± 0.01
	21	0.97^bD^ ± 0.01	0.97^bD^ ± 0.01	0.94^aD^ ± 0.01
	28	0.98^bE^ ± 0.01	0.99^bD^ ± 0.01	0.96^aE^ ± 0.01
Syneresis, %	1	60.01^aA^ ± 0.40	61.66^bC^ ± 0.12	61.94^bA^ ± 0.82
	7	61.40^aB^ ± 0.46	61.09^aB^ ± 0.26	63.64^bB^ ± 0.31
	14	58.72^aA^ ± 0.15	59.12^bA^ ± 0.16	62.09^cA^ ± 0.41
	21	62.06^bB^ ± 0.38	61.37^aB^ ± 0.07	64.14^cC^ ± 0.07
	28	63.16^bC^ ± 0.38	62.34^aD^ ± 0.07	65.11^cD^ ± 0.07
*L*	1	89.39^cBC^ ± 1.22	84.90^bA^ ± 0.38	82.89^aA^ ± 1.26
	7	88.56^cA^ ± 0.25	85.42^bB^ ± 0.22	83.86^aA^ ± 0.34
	14	90.71^cC^ ± 0.59	86.01^bC^ ± 0.79	83.29^aA^ ± 0.68
	21	88.37^cA^ ± 0.35	85.08^bC^ ± 0.16	83.28^aB^ ± 0.20
	28	89.17^cB^ ± 0.35	86.02^bC^ ± 0.16	84.22^aB^ ± 0.20
a*	1	−1.53^cB^ ± 0.14	−1.19^bC^ ± 0.05	0.72^cC^ ± 0.26
	7	−1.71^aA^ ± 0.06	−1.78^aA^ ± 0.18	0.44^bB^ ± 0.06
	14	−1.63^aB^ ± 0.04	−1.80^aA^ ± 0.13	0.22^bA^ ± 0.13
	21	−1.67^aB^ ± 0.01	−1.61^aB^ ± 0.06	0.54b^BC^ ± 0.06
	28	−1.68^aB^ ± 0.01	−1.66^aB^ ± 0.06	0.56^bC^ ± 0.06
b*	1	9.01^aA^ ± 0.61	34.79^bA^ ± 0.04	64.01^cA^ ± 0.44
	7	9.25^aB^ ± 0.01	35.60^bB^ ± 0.16	68.69^cC^ ± 0.42
	14	9.01^aA^ ± 0.12	35.26^aBC^ ± 1.18	67.98^cB^ ± 0.06
	21	9.72^aD^ ± 0.05	36.43^bC^ ± 0.11	68.17^cB^ ± 0.16
	28	9.62^aC^ ± 0.01	35.43^bB^ ± 0.13	68.27^cB^ ± 0.11
*C*	1	9.14^aA^ ± 0.62	34.80^bA^ ± 0.03	64.01^cA^ ± 0.43
	7	9.40^aB^ ± 0.01	35.64^bB^ ± 0.16	68.70^cC^ ± 0.43
	14	9.16^aA^ ± 0.12	35.31^bBC^ ± 1.19	67.98^cB^ ± 0.06
	21	9.87^aC^ ± 0.05	36.46^bC^ ± 0.12	68.17^cC^ ± 0.16
	28	9.81^aC^ ± 0.05	36.56^bC^ ± 0.12	68.70^cC^ ± 0.16
*h*	1	99.59^cA^ ± 0.26	91.97^bA^ ± 0.08	89.36^aA^ ± 0.23
	7	100.50^cB^ ± 0.36	92.86^bBC^ ± 0.29	89.63^aB^ ± 0.06
	14	100.24^cB^ ± 0.23	92.93^bC^ ± 0.11	89.82^aC^ ± 0.11
	21	99.76^cA^ ± 0.09	92.53^bB^ ± 0.08	89.54^aB^ ± 0.05
	28	100.16^cB^ ± 0.03	92.83^bC^ ± 0.06	89.84^aC^ ± 0.02
Yellowness index	1	22.24^aC^ ± 0.72	60.82^bC^ ± 0.99	94.45^cA^ ± 0.82
	7	22.25^aC^ ± 0.93	61.82^bA^ ± 0.58	102.13^cB^ ± 0.28
	14	20,59^aAB^ ± 0.95	61.24^bA^ ± 1.40	114.95^cC^ ± 1.50
	21	21.95^aB^ ± 0.18	62.42^bB^ ± 0.10	96.67^cA^ ± 0.29
	28	20.95^aA^ ± 0.18	62.12^bB^ ± 0.11	96.17^cA^ ± 0.25

The degree of syneresis in experimental yogurt samples increased during 28 days of storage: for the control sample—from 60.01% ± 0.40 to 63.16% ± 0.38; for TE—from 61.66% ± 0.12 to 62.34% ± 0.07 and for N—from 61.94% ± 0.82 to 65.11% ± 0.07. The addition of TE insignificantly reduced the degree of syneresis, while N increased it, which is related to its effect on the stratification of the milk clot.

The results of the chrominance study indicated that the control sample had a higher brightness than the other yogurt samples and a green color (negative a* values). The addition of TE reduces the intensity of the green color, and N shifts it toward the red (positive a* values). All samples have a yellow color. Saturation and color stability were the lowest for the control sample and amounted to 9.81 ± 0.05 units at the end of the storage period. This indicator significantly increased with the addition of curcumin preparations: for TE, the maximum value reached 36.56 ± 0.12, and for N—68.70 ± 0.16. For the h, a slight decrease was observed for the samples TE and N, compared to the control. The results of the measurement of the yellowness index are consistent with the data for (b*). For the control, its decrease was observed during storage, while for TE, it increased from 60.82 ± 0.99 to 62.12 ± 0.11 and for N from 94.45 ± 0.82 to 96.17 ± 0.25.

Explanatory note: physicochemical indicators of yogurt samples during 28 days of storage, where the following subscript mean: L* is lightness (from 0—222 black to 100—white), a* is color from red (+) to green (−), b* is color from yellow (+) to blue (−), *C* is color purity, and intensity and *h* is color hue.

**Figure 2:**
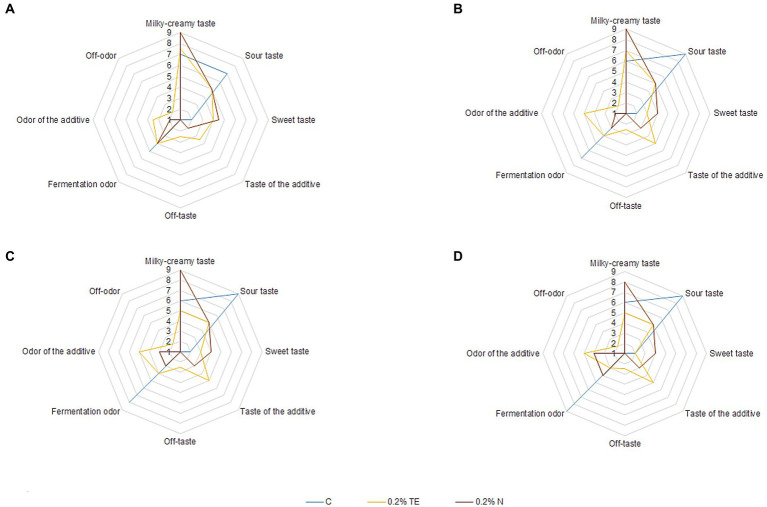
Sensory evaluation of yogurt sample. **(A)** 7th day, **(B)** 14th day, **(C)** 21st day, and **(D)** 28th day.

Sensory evaluation of experimental samples of yogurt was carried out on the 7th, 14th, 21st, and 28th days of storage (Figure 2).

From the obtained data, it can be seen that the most pronounced sour-milk taste was observed for the control sample, which became more saturated during storage, while the addition of both curcumin preparations slightly reduced its intensity, especially for N during the first 3 weeks, but at the end of the storage period, it was the same for both N and TE, which correlates with the data on the amount of lactic acid formed in the experimental samples. The sour-milk aroma in the case of TE application was greater than that of N during the first 3 weeks, but at the end of the storage period, it was barely perceptible. Thus, for TE, already on the 7th day, the aroma of the additive was observed, which increased during the 28 days of storage, which is not a characteristic feature of yogurt (42). At the same time, for N, the aroma of the additive after 1 week of storage was unobtrusive and began to intensify after the second week of storage for yogurt samples. It should be noted that TE significantly affected the taste of yogurt and generally began to deteriorate after the first week of storage due to the sensation of an extraneous bitter aftertaste. For N, the aftertaste of the additive was barely perceptible and did not affect the overall taste perception even at the end of the storage period. Moreover, N imparted a sweet taste and a rich, creamy flavor to the yogurt, which remained stable until the end of storage, while for TE, its intensity decreased to the control level or below. It is necessary to note the property of N to impart a sweet taste and effectively imitate the taste of milk fat, which is extremely attractive to consumers (43). At the same time, an unusual aftertaste was detected for the TE sample on the 28th day, which may be related to the formation of insoluble sediment of the additive that settled over time. For N, the aftertaste was barely pronounced at the end of the storage period, which is also an advantage of the dispersed form of curcumin and practically does not form sediment. Thus, according to the results of sensory evaluation, the best sample was with 0.20% N, the organoleptic properties of which did not deteriorate during the 28 days of storage. For TE, taste deterioration was noted already on the 7th day of storage, which limits the storage period and does not make it possible to achieve the set technological result. Moreover, TE partially dilutes the yogurt structure, which was observed on the 28th day of storage.

### 3.4. Sedimentation

As can be seen from Table 4, the lower the difference between the spectrophotometer values of TE and N aqueous solutions, the lower the sedimentation. Thus, for TE after centrifugation, the value decreases from 0.95 ± 0.02 to 0.028 ± 0.002, and for N from 0.44 ± 0.03 to 0.37 ± 0.02. Sedimentation for TE is significantly higher than N and is 97.1 and 15.9, respectively. High sedimentation is a technological obstacle that significantly limits the applicability of TE in the production of yogurt and dairy products, which will undoubtedly affect the sensory indicators of yogurt with these additives. Sensory analyses are described in the corresponding section of this article.

**Table 4:** Sedimentation ability of curcumin additives.

Sample	Spectrometer values		Sedimentation, %
	Before centrifugation	After centrifugation	
TE	0.95^bB^ ± 0.02	0.03^aA^ ± 0.02	97.10
N	0.44^bA^ ± 0.03	0.37^a^ ± 0.02	15.90

## 4. Discussion

### 4.1. Antimicrobial activity

In one of the first works devoted to the antibacterial activity of curcumin, the authors reported on the pronounced effect against G^+^ bacteria (*S. aureus*, *S. epidermidis*, and *Streptococcus pyogenes*) and GP^−^ bacteria (*Acinetobacter lwoffii* and *Alcaligenes faecalis*) (44). The mechanism of curcumin’s inhibition of antibacterial activity is that it inhibits the formation of biofilms by pathogenic microorganisms (45). Unlike many antibiotics, it does not kill the bacteria themselves and does not destroy their biofilm but inhibits the process of its formation due to numerous interactions with molecular targets and transduction pathways due to the antibacterial mechanism (46). Despite various studies on the antibacterial and antifungal activity of curcumin, there is still a lack of data in the scientific literature on the effect on a wide range of strains, and the effect of bioavailable forms of curcumin is poorly studied, delineating the scope of scientific interest.

N showed higher antimicrobial activity against G^−^ bacteria (*P. aeruginosa* and *Y. enterocolitica*), which might be attributed to a stabilizing effect of the formulation, based on GRAS additives, which to some extent modifies the properties of curcumin, in particular antibacterial ones (47). TE has pronounced antifungal activity against various strains of Candida, which is consistent with previously published results (48).

The antifungal activity of N is slightly lower than for TE in the case of *P. chrysogenum, P. brevicompactum* and higher in *P. italicum, P. aurantiogriseum*, a lower profungicidal capacity of curcumin in its bioavailable form than its antibacterial capacity, which was attributed to a higher degree of penetration into the bacterial cell (49). N, with increased water, is a bioavailable form of curcumin, which can be an effective and natural preservative for use in food technology. Due to the unique properties of the formulation, N is able to inhibit the growth of food pathogens, such as *E. coli*, *S. aureus*, *B. subtilis*, *B. cereus*, *Y. enterocolitica*, and *Penicillium notatum*.

### 4.2. Microbiological analyses

Natural food additives with nutraceutical properties are attractive to food manufacturers, but their presence in fermented milk products can affect the growth and development of lactic acid bacteria in different ways (50). Additives that have a pronounced antimicrobial effect and can act as a natural preservative are of particular interest, as they can limit or completely eliminate the use of artificially synthesized substances. Tay Abdelrazik and Fouad M. F. Elshaghabee (2020) found that the form of curcumin enabled to maintain the number of viable cells of *Lactobacillus plantarum* EMCC 1027 on the 20th day of storage in fermented soy milk at the level of (8.20 ± 0.91) log CFU/g, and in cow’s milk at the level of (7.10 ± 0.85) log CFU/g. In our experiment, it was determined that the form of curcumin (NOMICU® L-100) in fermented cow’s milk allows ensuring this indicator on the 28th day of storage at the level of 7.7 log CFU/g, while the results of the effect of TE on the survival of the probiotic during the 21st day are comparable to the generally known scientific data. The synergistic effect of curcumin with probiotic cultures was also noted by other scientists (51), who showed consistency in the number of probiotic microorganisms with the recommended level (10^6^ CFU/ml) at the end of the storage period.

There are also studies available on the use of nanoemulsions of natural functional ingredients to support the development of probiotic cultures and secure the recommended level of 6 log CFU/g during the entire storage period (52). Sharma et al. (53) confirmed the ability of curcumin to significantly reduce the growth of yeast and mold in functional dairy, which was observed in this experiment. The more pronounced antimicrobial effect of N than for TE is a consequence of its specific formulation enabling the release of curcumin on the surface of the bacterial cell and into its membrane, which enables it to exhibit an inhibitory effect with the formation of a bacterial biofilm (45, 54). In our study, during 28 days of storage, no coliforms were detected (both for TE and N). These results confirm the antimicrobial activity of curcumin and justify its use in food products where the number of coliforms should not exceed <100 CFU/g (Fung., 2009).

Turmeric extract (2.0%) is known to have a positive effect on the growth of *L. rhamnosus* GG and *Bifidobacterium animalis* subsp. *lactis* BB-12 (55). However, as the dose of the supplement decreases, its ability to support vital activity also decreases or is not observed at all at extremely low concentrations (0.01%) (56). At the same time, TE does not significantly stimulate the activity of *Lactobacillus* sp. (*L. acidophilus* A001F8, *L. rhamnosus* A001G8, *L. paracasei* A002C5, *L. plantarum* A003A7, and *L. casei* A003D4) (56), what was established during our study. Such a fenomena may be related to the fungicidal effect of curcuminoids, which could suppress the vital activity of such cultures as *Streptococcus thermophilus*, *Lactobacillus* *delbrueckii* subsp. *bulgaricus*, YF-L812, *Bifidobacterium* animalis subsp. *lactis* BB-12, and others (57). Yazdi et al. reported that TE ensures the growth of *Lactobacillus rhamnosus* GG and *Bifidobacterium animalis* subsp. *lactis* BB-12 bacteria even after 72 h of their development (58). The positive effect of N on the microorganisms *Lactobacillus delbrueckii* subsp*. Bulgaricus, Streptococcus thermophilus, Lactobacillus acidophilus, Lactobacillus johnsonii,* and *Lacticaseibacillus casei* in our analysis may be related to the better dispersibility and thus higher bioavailability of the curcumin that might support the vital activity of probiotics compared to the crude curcumin extract.

### 4.3. Indicators of quality

Determination of the acidity of fermented milk products is a prerequisite for the development of products with technological and functional additives (59), which includes curcumin. One of the most important parameters is the determination of the additive’s effect on the physicochemical parameters of the product during its storage. The increase of the acidity in the experimental samples of yogurt matches the level of the control sample, which indicates the formation of lactic acid and is one of the preservative factors in fermented milk products (60). Khanji et al. (61) proved that curcumin does not affect the process of acid formation and vital activity of *Lactobacillus delbrueckii* subsp. *bulgaricus* and *Streptococcus thermophilus*. Guerra et al. (41) also reported that curcumin extract did not significantly affect titrated and active acidity values. Seham et al. (62) determined that curcumin extract significantly increases active and titratable acidity, especially after the 9th day of storage, which the authors attribute to the use of *B. bifidum* ATCC 15696*, B. bifidum* ATCC 29521 and *B. longum* as starter cultures.

The somewhat reduced degree of syneresis for TE may be due to the swelling of curcumin particles, which strengthens the bonds between the milk gel network (63). However, Guerra et al. (41) reported a significant increase in the degree of yogurt syneresis on the 21st day of storage, which is due to the destruction of the three-dimensional gel due to a high concentration of the additive (64). At the same time, in our study, a slight increase in the degree of syneresis for N was observed, which is noticeable at the end of the storage period. However, the destruction of the yogurt structure was not observed because the dose used was much lower than in the predecessors.

The greater brightness of the control sample and the presence of a green color (negative values of a*) may be due to a combination of factors, such as the presence of riboflavin in milk used for the production of yogurt samples (65), oxidation of fatty acids (66), the proteolytic activity of yogurt (41). Positive (b*) values for yogurt samples with curcumin support the suggestion that this additive may be used as a natural colorant (67). However, the decrease in h for yogurt samples suggests that curcumin as a colorant may also have some limitations due to some instability. That is why it is important to determine the rational dose of curcumin and the scientific substantiation of the composition of yogurt with it, which will allow obtaining a product with predicted quality indicators. Almeida et al. (68) also noted the deterioration of color uniformity of products with curcumin during storage. The increase in the yellowness index for yogurt samples has to be attributed to the natural color of this compound (69). In our study, we showed that N formulation enables maintaining the amount of lactic acid at the same level as in the control sample, which certainly shows a preservative effect. It should be noted that this additive improves the color indicators of the product, which increases its attractiveness to the consumer. Sensory evaluation of experimental yogurt samples during 28 days of storage confirmed that N (0.2%) provided the best taste and aroma indicators.

Some researchers (25) suggested that fermented dairy products are the better delivery system for curcumin. Considering that most experiments on the sensory evaluation of products with curcumin were conducted specifically to determine color parameters (41, 70, 71), there is still limited data and certain contradictions in the scientific literature regarding its effect on other sensory indicators (taste, aftertaste, aroma, odor, etc.) of fermented milk products, which determines the authors’ interest in conducting such a study. Deterioration of the aroma and odor of yogurt was reported when the dose of TE exceeded 1.25% (41), whereas, in our study, this effect was already observed at doses of 0.20% and may be related to the different composition of the extract. Тhe partial dilution observed at the end of the storage period for TE may be related to the chemical structure of curcumin. The balance of stability in yogurt is achieved by the attraction and repulsion forces (41). Curcuminoids may generate the breakdown of the gel structure of the yogurt in the last days of storage in samples with the highest concentrations of curcumin.

Another study noted the ability of TE to impart a spicy flavor to yogurt (72). In this experiment, a slightly bitter taste was observed that increased with TE concentration, while N imparted a sweet aftertaste to the product. Lim et al. (73) attributed the bitter taste from the addition of curcumin to its terpenoid content, while Drewnowski and Gomez-Carneros C (74) attributed it to volatiles and phenolic compounds, limiting its dosage for formulation use different products. Britto et al. (75) reported the appearance of a sour taste in yogurts with curcumin. However, we did not observe this, which is probably due to the minimal dosage of curcumin preparations used in our experiment and the high purity of the curcuminoid extract.

The positive effect of encapsulated curcumin in a dairy food system with a low-fat content was noted by various scientists who pointed to its ability, in the absence of fat, to form connections with micelles of milk proteins (76). Moreover, curcumin formulation, unlike the crude curcumin extract, improves the purity of the taste and helps to reduce the expense of the production technology (77).

### 4.4. Sedimentation

Szterk et al. (78) reported that high sedimentation is a major shortcoming of most curcumin-based preparations. This is partially explained by the high content of curcuminoids (over 90%), which is also confirmed in our research because TE contains approximately 95% curcuminoids, while N is approximately 25%–35%. However, the reduced content of curcuminoids itself does not guarantee low sedimentation. Scientists indicate that the reduction of sedimentation is also influenced by the composition of the preparation, in particular, the components that ensure its bioavailability. In addition, TE is characterized by a low bioavailability of curcuminoids due to their insolubility in water. Turmeric extract also tends to crystallize over time (79). Such characteristics limit the use of traditional curcuminoid additives in the industrial production of food products, in particular dairy, since the formation of an insoluble precipitate and its sedimentation can not only affect the production process itself but also worsen the visual perception of the consumer, and lead to numerous defects, such as syneresis of fermented milk drinks, deterioration of taste due to the appearance of a bitter aftertaste, and others (41).

### 4.5. Possible medical implications

In the last decades, there have been many attempts to enrich different types of food with natural products with beneficial properties (80). It is especially important nowadays, in a post-pandemic time, with even 70% of the population being overweight and obese, with gut microbial dysbiosis, and being at a high risk of chronic diseases, including the two largest killers, cardiovascular diseases and cancers, that together are responsible for even 30 million deaths per year (81). Unfortunately, most of the food products available on the market do not contain natural products with confirmed efficacy and safety or contain them in insufficient amounts. It also refers to dairy products, which may have beneficial effects on all-cause and cause-specific mortality (82). Therefore, there is a large need to look for functional foods/supplements that may effectively enrich dairy products and make them healthier for the general population, as well as those with specific indications. Our unique preparation of curcumin, which is highly purified and dispersible, and characterized by high bioavailability, gives a chance to start the line of products, which on the one hand, due to its composition (fermented dairy products), may reduce by themselves morbidity and mortality risk. A suitable formulation of curcumin might significantly enhance these beneficial properties. Curcumin, due to its recognized anti-inflammatory and antioxidant properties, may have significant effects on patients at risk of cardiovascular diseases (83), and in addition, due to its antimicrobial effects, may also be an effective and safe option for improving COVID-19 disease outcomes (84).

## 5. Conclusion

1. NOMICU® L-100 formulation (N) shows antimicrobial activity against pathogenic microorganisms, with the highest activity observed against G^+^ bacteria (*S. pneumoniae*) and fungi (*P. italicum* and *P. aurantiogriseum*).

2. Based on the conducted microbiological analysis of yogurt samples with TE and N (mass fraction −0.1% to 0.25%) on the 7th, 14th, 21st, and 28th day of storage, it was established that these additives show not only antibacterial activity but also support the development and vital activity of the probiotic culture *Bifidobacterium animalis* subsp. *lactis* BB-12. For N, the amount of probiotics was at the level recommended for functional fermented milk drinks (7–9 log CFU/g) throughout the entire storage period.

3. Evaluation of the quality indicators of yogurt with N (0.2%) indicates that the formation of lactic acid occurs at almost the same level as in the control sample. It should be noted that this additive improves the color indicators of the product, which increases its attractiveness to the consumer. Sensory evaluation of experimental yogurt samples during 28 days of storage confirmed that N (0.2%) provided the best taste and aroma indicators.

4. Thus, the use of N (0.2%) due to its antibacterial properties, as well as the ability to support the vital activity of probiotic microorganisms, allows for avoiding the use of other preservatives in the yogurt formulation. Unlike crude turmeric extract (TE), N has excellent properties to enhance the taste of milk fat and give the product an attractive color, which makes the fermented dairy product even more attractive to consumers.

## Data availability statement

The original contributions presented in the study are included in the article/supplementary material, further inquiries can be directed to the corresponding authors.

## Author contributions

MB-O and JU: conceptualization. AM and MK: methodology. AZ-P: software. MB-O, MB, and JU: validation and supervision. MB: formal analysis. MB-O, AM, MK, and PB: investigation. AM: resources. MB-O: data curation and visualization. MB-O, AM, JU, and MB: writing of original draft preparation. MB and MK: writing of review and editing. JU: project administration and funding acquisition. All authors contributed to the article and approved the submitted version.

## Funding

The research project was carried out with the support of the funds of the Polish National Center for Research and Development, under the framework program 1.3.1. The technology for extending the shelf life of probiotic-nutraceutical dairy products is protected by patent application P.443720 filed by Dairy Biotechnologies Ltd.

## Conflict of interest

Author JU was employed by the company Dairy Biotechnologies Ltd. The remaining authors declare that the research was conducted in the absence of any commercial or financial relationships that could be construed as a potential conflict of interest.

## Publisher’s note

All claims expressed in this article are solely those of the authors and do not necessarily represent those of their affiliated organizations, or those of the publisher, the editors and the reviewers. Any product that may be evaluated in this article, or claim that may be made by its manufacturer, is not guaranteed or endorsed by the publisher.
